# Efficacy of a Multicomponent Occupational Therapy Intervention on Balance, Functional Mobility, and Proprioception in Institutionalized Older Adults: A Randomized Controlled Pilot Trial

**DOI:** 10.3390/healthcare13182287

**Published:** 2025-09-12

**Authors:** Alejandro Caña-Pino, Alba Marín-Rubio

**Affiliations:** 1 Surgical Medical-Therapy Department, Medicine Faculty and Health Sciences, University of Extremadura, 06006 Badajoz, Spain; amarinru@alumnos.unex.es; 2 Research Group PhysioH (Fisioterapia e Hipoterapia), University of Extremadura, 06006 Badajoz, Spain

**Keywords:** occupational therapy, fall prevention, functional mobility, sensorimotor training, aging population

## Abstract

**Background**: With the progressive aging of the in institutionalized settings population, functional decline—manifested as reduced proprioception, joint stiffness, and muscle loss—poses a growing threat to the autonomy and quality of life of older adults. Occupational therapy plays a central role in addressing these challenges through targeted, evidence-based interventions. **Objectives:** This clinical trial evaluates the effectiveness of a multicomponent occupational therapy intervention that integrates balance and postural stability exercises, proprioceptive stimulation, and lower-limb strengthening with conventional gerontogymnastics. The program was designed to improve gait performance, reduce fall risk, and promote independence in institutionalized older adults. **Methods:** A total of 35 community-dwelling older adults were randomly assigned to three groups: gerontogymnastics intervention (n = 13), multicomponent intervention (n = 13), and control (n = 9). Participants underwent a 6-week intervention comprising two 45 min sessions per week. Pre- and post-intervention assessments focused on postural stability and balance-related functional outcomes. **Results:** The multicomponent group exhibited trends toward improvement in balance, proprioception, and functional mobility, although these did not reach statistical significance. Clinically meaningful improvements were defined using minimally clinically important differences (MCIDs) for functional measures such as Timed UP and Go (TUG) (>1.3 s) and Performance-Oriented Mobility Assessment (POMA) (≥3 points). The multicomponent group showed a 22.1% improvement in proprioceptive accuracy and a 13.9% improvement in mobility (TUG). Additionally, this trend may indicate a potential protective effect against age-related functional decline. **Conclusions**: These findings suggest that a multicomponent occupational therapy intervention may help maintain mobility and reduce functional decline in institutionalized older adults. Statistically significant gains were observed in lower-limb strength, while other improvements—such as proprioception and balance—did not reach significance but approached clinical relevance. These preliminary results support further investigation into balance-specific training within occupational therapy to promote independence and reduce fall risk. Interpretation should remain cautious due to the small sample size (n = 35) and short intervention duration, which limit statistical power and generalizability.

## 1. Introduction

Across many countries, older adults over 65 years now represent more than 20% of the total population, reflecting the global demographic shift toward aging. This phenomenon presents significant challenges and opportunities for public health, long-term care, and quality of life. For example, in Spain, adults over 65 account for 20.4% of the population. According to Spanish data, approximately 30–40% of institutionalized older adults experience at least one fall annually [1,2,3,4], leading to hospitalization in 5–10% of cases and contributing significantly to healthcare costs and loss of autonomy. Such fall rates align with global data indicating 2.4 falls/person-year in institutionalized adults and major risk associations like comorbidity and polypharmacy [5,6]. From a biological perspective, aging is a progressive process characterized by the gradual accumulation of molecular and cellular damage. These alterations impair biological components by reducing their capacity for repair, stress adaptation, and internal physiological regulation (homeostasis) [7]. Clinically, this deterioration manifests as a progressive decline in both physical and cognitive abilities, increasing vulnerability to age-related conditions such as osteoporosis, osteoarthritis, and sarcopenia [8,9,10,11]. At the musculoskeletal level, this process involves a decrease in muscle mass and an increase in body fat, contributing to a gradual reduction in muscular strength and the ability to maintain balance [12,13].

Key functional components such as balance (the observable ability to maintain the body’s center of mass within the base of support), proprioception, and underlying postural control mechanisms play a critical role in maintaining mobility and independence, enabling older adults to perform daily activities safely and efficiently. Decline in these areas is directly linked to an increased risk of falls, injury, and functional dependency, particularly among institutionalized populations [1,2,14,15,16].

Neurological changes associated with aging also impair motor coordination and responsiveness to external stimuli, further compromising postural stability [14]. As the neuromuscular system deteriorates, the ability to maintain postural stability in activities of daily living decreases significantly, with previous studies reporting up to a 30% decline in balance performance and gait stability between the ages of 65 and 80, particularly due to losses in proprioceptive acuity, lower-limb strength, and reaction time [17,18]. These deficits significantly elevate the risk of falls, which are a major cause of morbidity, disability, and loss of independence in older adults. For example, in Spain, over 323,000 older adults currently reside in long-term care facilities, reinforcing the need for effective intervention programs that promote physical autonomy—defined as the individual’s ability to independently carry out essential daily activities such as mobility, self-care, and social engagement—and prevent progressive functional decline [19]. Gerontogymnastics refers to a structured program of low-impact physical exercises for older adults, typically including rhythmic seated and standing movements, gentle stretching, coordination drills, and flexibility routines. These activities are tailored for age-related capacities and delivered under professional supervision in a preventive framework [20]. Although gerontogymnastics remains a useful preventive strategy, its effects may be limited without the integration of sensorimotor stimulation and targeted balance training [21,22]. Recent studies have shown that multicomponent exercise programs—incorporating balance, postural stability, strength, and mobility training—are more effective than single-modality interventions in improving functional outcomes and reducing fall risk in older adults [1,18,23,24,25,26]. Recent meta-analyses demonstrate that multicomponent exercise significantly improves strength [standardized mean differences (SMD ≈ 0.31), balance (SMD ≈ 0.27), and Timed UP and Go (TUG) performance (SMD ≈ −3.05 s) in frail older adults [27]. Network meta-analysis also ranks balance-focused training highest for fall prevention (RR reduction ~23–34%) [28]. In this sense, gerontogymnastics incorporates low-impact physical exercises aimed at maintaining mobility and flexibility. When combined with proprioceptive and balance-specific training, it enhances neuromuscular control and functional autonomy, key to postural stability and fall prevention. Unlike standard physical exercise or physiotherapy programs, gerontogymnastics is specifically tailored to meet the physiological and functional needs of older adults. It emphasizes repetitive, low-intensity movements that integrate balance, joint mobility, and muscle coordination within a preventive and non-clinical framework [29]. While gerontogymnastics may indirectly influence balance through general mobility and coordination activities, it does not include targeted proprioceptive or postural control tasks. In contrast, the multicomponent intervention integrates specific exercises designed to challenge postural stability and enhance neuromuscular feedback mechanisms. In this study, we use ‘balance’ to refer to the observable performance of maintaining the body’s center of mass within the base of support, assessed through clinical tests such as the POMA Performance-Oriented Mobility Assessment (POMA). In contrast, we use ‘postural stability’ to denote the dynamic functional expression of postural control mechanisms, particularly the neuromuscular capacity to resist or recover from perturbations [30,31]. For clarity, postural stability is considered here as a measurable proxy of postural control, whereas balance represents the broader functional outcome [18,31].

In this sense, postural stability refers specifically to the dynamic control of body position during movement, including the ability to resist perturbations or regain balance. Functional decline in older adults is not only characterized by impaired balance, but also by diminished gait performance, muscle strength, and proprioception. Gait is a complex, dynamic function highly sensitive to neuromuscular deficits and a major predictor of falls and loss of independence [18]. Muscle strength, particularly in the lower limbs, is essential for initiating and maintaining posture and mobility [13]. Similarly, proprioceptive deficits compromise sensorimotor integration, reducing one’s ability to detect joint positioning and maintain stability during movement [30].

In occupational therapy, there is increasing emphasis on designing interventions that are functionally meaningful, preventive in nature, and evidence-based [24]. Such programs should be repetitive, task-specific, and grounded in principles of motor relearning and neuroplasticity [32]. However, evidence on the effectiveness of multicomponent interventions in institutionalized older adults, especially in combination with traditional gerontogymnastics programs, is still limited [33]. This lack of studies in institutionalized older adults reinforces the need to generate local data to support the design of more effective therapeutic programs. Despite the growing elderly institutionalized population in Spain, few studies have evaluated multicomponent interventions tailored to improve proprioception, gait, and postural stability in this specific context.

The variables assessed in this study—balance, proprioception, functional mobility, muscle strength, and perceived exertion—are interconnected components of the sensorimotor system that jointly influence fall risk. Postural stability represents the observable outcome of postural control mechanisms, which integrate sensory input (notably proprioception) with motor responses (e.g., muscular contractions and gait adaptations). Deficits in any of these components can cascade into reduced balance confidence, slower mobility, and greater risk of falls. In this study, we designate balance, proprioception, and mobility [POMA, Joint Position Sense (JPS), and TUG] as primary outcomes due to their direct clinical relevance to fall prevention. Secondary outcomes such as lower-limb strength and perceived exertion are considered mediators of functional capacity and are key to interpreting changes in the primary variables. Balance was assessed using the POMA, a validated tool for evaluating fall risk. Functional mobility was measured using the TUG test, which captures dynamic mobility. Proprioceptive acuity was examined through JPS tasks, reflecting neuromuscular control. Functional independence was assessed using the Functional Independence Measure (FIM), widely applied in rehabilitation contexts.

This clinical trial aims to evaluate the effectiveness of a multicomponent occupational therapy intervention—combining targeted exercises for balance, proprioception, and lower-limb strength with conventional gerontogymnastics—in institutionalized older adults. The primary outcomes assessed include balance performance (POMA), functional mobility (TUG), functional independence (FIM) and proprioceptive acuity (JPS), while secondary outcomes included lower-limb muscle strength, and perceived exertion (Borg scale). The intervention is compared to a traditional gerontogymnastics-only protocol. This therapeutic approach is grounded in occupational therapy models such as the Model of Human Occupation (MOHO), emphasizing volition, performance skills, and engagement in meaningful, occupation-based activities to support healthy aging and fall prevention.

We hypothesize that multicomponent intervention will produce clinically meaningful improvements in balance-related functional performance and may serve as a protective factor against physical decline. Improvements were considered clinically meaningful if they met or exceeded established MCID thresholds: a reduction of ≥3 s in TUG, ≥3 points in POMA, and ≥2° in JPS error [34,35,36,37,38,39]. This integrative approach seeks to establish itself as a viable therapeutic strategy to promote autonomy, reduce fall risk, and support healthy aging in residential care settings. It is suggested that the intervention could be a replicable model in other residential settings, integrating occupational therapy principles based on functional performance and meaningful participation.

## 2. Methods

### 2.1. Design

This study was a randomized controlled pilot trial aimed at evaluating the effects of different interventions in institutionalized older adults. Participants were randomly assigned to one of three groups to enhance internal validity by reducing selection bias and balancing known and unknown confounding variables across groups. The randomization was performed using a computer-generated random sequence, and allocation concealment was ensured using sealed opaque envelopes. This study was conceived as a pilot trial; therefore, no formal sample size calculation was performed. Participant recruitment was based on feasibility and institutional availability. Ethical approval was granted by the Ethics Committee of the University of Extremadura, Spain (Project Code: 46/2025; Approval Date: 29 January 2025), and the study was registered at ClinicalTrials.gov (Identifier: NCT06844578). As such, no a priori power calculation was performed. The sample size was based on feasibility and the number of eligible participants available at the collaborating institution. The results should therefore be interpreted as preliminary and exploratory, providing effect size estimates to inform future adequately powered trials. This study was conducted as a randomized controlled trial (RCT) and reported in accordance with the CONSORT 2010 statement for parallel-group trials.

All assessments and interventions were conducted between January and April 2025. To prevent bias, different researchers were responsible for the evaluation and the implementation of the intervention protocols. Outcome assessments were conducted by a physiotherapist blinded to group allocation to reduce evaluation bias.

### 2.2. Participants and Randomization

A total of 78 residents were initially assessed for eligibility. Of these, 60 met preliminary inclusion criteria and were considered the potential sample for further screening at the Nuestra Señora del Rosario Care Home in Cáceres, Spain. After eligibility screening based on predefined inclusion and exclusion criteria, 35 participants were selected. Inclusion criteria were: age ≥ 65 years, institutionalized status, ability to maintain upright standing without continuous assistance, and a POMA score <25, indicating balance impairment. Exclusion criteria included contraindications for physical activity, wheelchair dependence, severe cognitive impairment (MMSE < 10), or intense pain (VAS > 4). All participants gave written informed consent.

Participants were randomized into three groups: Intervention Group 1 (IG1) (n = 13, conventional gerontogymnastics program), Intervention Group 2 (IG2) (n = 13, multicomponent program combining gerontogymnastics, proprioceptive stimulation, and strength training) and Control Group (CG3) (n = 9, no therapeutic intervention). Although participants were randomly assigned to the three groups, gender was not used as a stratification factor due to the limited sample size. The final sample consisted of 35 institutionalized older adults: 13 participants in IG1, 13 in IG2, and 9 in CG3. Regarding sex distribution, IG1 included 6 males and 7 females, IG2 included 5 males and 8 females, and CG3 included 4 males and 5 females.

**Figure 1:**
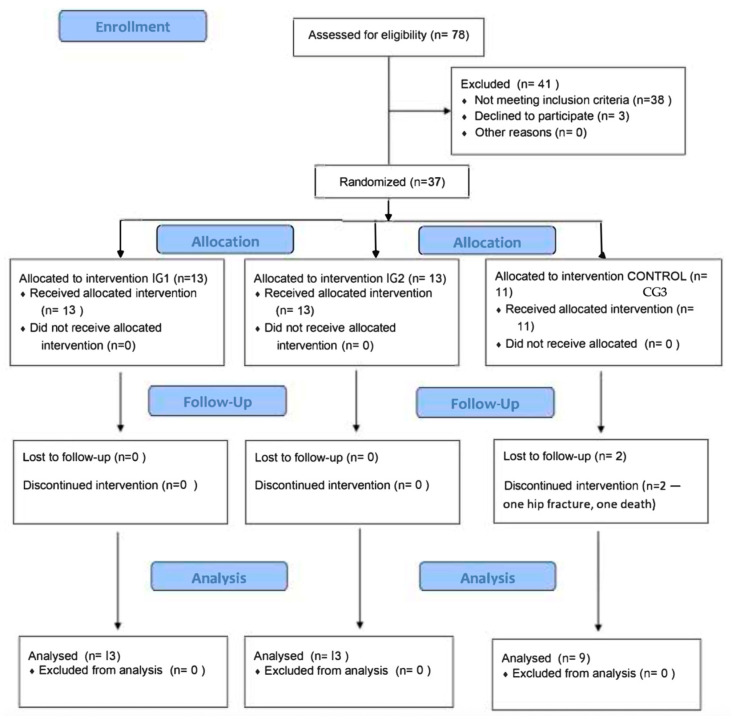
Flowchart of this study.

The control group (CG3) received standard institutional care without additional exercise interventions during the study period. The recruitment process and participant flow through the study are detailed in the CONSORT flow diagram (Figure 1).

### 2.3. Outcome Measures

Outcome variables were pre-specified and classified as primary or secondary prior to data analysis, as recommended by CONSORT. These variables were selected based on clinical relevance and sensitivity to change in institutionalized older adults. The study variables were organized into three categories:

1. *Descriptive variables*: age, height, body mass, Mini-Mental State Examination (MMSE), baseline pain (Visual Analog Scale).

Primary outcomes comprised balance performance (POMA), functional mobility (TUG), functional independence (FIM), and proprioceptive acuity (JPS), while secondary outcomes included lower-limb muscle strength and perceived exertion (Borg scale).

2. *Primary outcomes*:
  - Balance: assessed with the POMA.
  - Functional Mobility: assessed with the TUG test.
  - Functional independence: assessed with the FIM.
  - Proprioception: measured via the JPS test.

3. *Secondary outcomes*:
  - Muscle strength: assessed using the Daniels scale for right quadriceps and hamstring.
  - Perceived exertion: evaluated with the modified Borg scale.

To accurately assess the effects of the interventions, standardized instruments were employed based on their proven validity and reliability in evaluating key physical and functional variables in older adults.

*MMSE:* A brief tool designed to assess cognitive status, particularly in older adults. It evaluates domains such as orientation, memory, attention, and language, with a maximum score of 30. Scores below 24 indicate significant cognitive decline [40]. Participants scoring above 10 were deemed cognitively suitable for the intervention, ensuring their understanding and participation.

*POMA*: This scale assesses both static and dynamic balance as well as gait in older adults. The total score ranges from 0 to 28, with lower values indicating higher fall risk and postural instability [41]. In this study, the POMA was selected for its strong reliability in detecting fall risk and tracking balance improvements in this population [42]. This scale has demonstrated high inter-rater reliability (ICC > 0.85) and construct validity in older adult populations for predicting fall risk and assessing gait and balance performance [41,43].

*Daniels and Worthingham Manual Muscle Testing*: Muscle strength was assessed by a trained evaluator using the Daniels and Worthingham Manual Muscle Testing method, which involves manual resistance applied by the evaluator to test the participant’s lower-limb strength through standardized procedures. Muscle strength is graded on a 0–5 scale, where 0 indicates no contraction and 5 represents normal strength against maximum resistance [44]. This scale was applied to assess key muscle groups involved in gait and postural stability, specifically the gluteus maximus, quadriceps, and hamstrings. Manual muscle testing per Daniels and Worthingham has shown acceptable inter-rater reliability in clinical settings, particularly for lower limb strength in older adults [45].

*VAS:* A psychometric tool used to quantify pain intensity. The scale ranges from 0 (no pain) to 10 (worst imaginable pain). Participants marked the point corresponding to their pain perception [46]. This scale assessed participants’ self-perceived general musculoskeletal pain during routine daily activities. Pain assessment was essential, as chronic pain may interfere with mobility-focused interventions in older adults [47].

*Modified Borg Scale:* A simplified version of the original Borg scale designed to measure perceived exertion during physical activity. It ranges from 0 (no exertion) to 10 (maximal exertion). This tool was used to monitor perceived effort during daily living activities before and after the intervention [48].

*FIM:* This scale evaluates functional independence across 18 items related to mobility, communication, activities of daily living (ADLs), hygiene, sphincter control, and locomotion. Scores range from 1 (total dependence) to 7 (complete independence). FIM is widely used in geriatric and rehabilitation contexts to track progress, tailor interventions, and quantify functional impact. The FIM has demonstrated high internal consistency (Cronbach’s α > 0.95), inter-rater reliability, and construct validity in institutionalized geriatric populations [49].

*TUG:* The TUG test is a widely used functional assessment to evaluate mobility and dynamic balance in older adults. Although it has been employed as a proxy for fall risk, evidence suggests that its predictive validity is limited if applied in isolation, and it should be interpreted in combination with other functional measures [34].

It records the time (in seconds) needed to stand up from a chair, walk three meters, turn around, return, and sit down [50]. Its predictive validity for future falls has been supported by systematic reviews and meta-analyses [34]. The test demonstrates excellent test–retest reliability (ICC = 0.99) and strong construct validity in evaluating functional mobility and fall risk in this population [38,50].

*JPS:* A proprioceptive assessment tool used to measure joint repositioning error. In this study, participants were assessed while standing, using an iPhone^®^ (Apple Inc, Cupertino, CA, USA) inclinometer aligned with the trunk at the hip level. Participants leaned 30° forward, memorized the position, and then attempted to reproduce it with eyes closed. The angular error in joint repositioning was recorded in degrees [39]. Proprioception evaluation was key to assess spatial awareness and motor coordination [30]. While proprioception is a foundational component of postural control, JPS does not fully represent the neurophysiological construct of postural control. In this study, JPS is used as a functional proxy for sensorimotor integrity relevant to postural stability. The use of smartphone inclinometers for assessing JPS has shown acceptable intra-rater reliability and concurrent validity for joint position error in older adults with and without chronic pain [39]. Its practicality, low cost, and portability make it a feasible tool for clinical use, particularly in institutional environments with limited access to laboratory-grade equipment.

### 2.4. Assessment Protocol

A qualified and independent physiotherapist conducted participant screening, strictly applying the predefined inclusion and exclusion criteria. This professional was not involved in any part of the intervention or post-intervention assessments, thus ensuring unbiased sample selection.

All assessments were conducted individually in a designated quiet room. The following order was used to minimize fatigue and interference effects: (1) MMSE, (2) VAS for pain, (3) Manual Muscle Testing (Daniels and Worthingham), (4) JPS test, (5) TUG, (6) POMA, and (7) FIM. Standardized instructions and rest periods were provided as needed between tests.

Functional assessments were conducted in two stages: a baseline evaluation (Week 0), prior to the intervention, and a final evaluation (Week 7), after program completion. A one-week post-intervention buffer was incorporated to schedule outcome assessments without interference from the training sessions, allowing standardized testing conditions across all groups. The aim was to compare physical and functional status across time points and identify potential intervention effects.

### 2.5. Intervention Protocol

The interventions were delivered entirely by an experienced occupational therapist, who implemented the assigned program for each participant. Session attendance and perceived exertion were logged systematically. The therapist adapted the sessions to individual capabilities and supervised all activities to ensure safety, proper execution, and adherence to treatment. Intervention fidelity was ensured by using a standardized protocol manual, and all sessions were monitored for adherence and safety. A structured intervention manual was followed for all sessions. Attendance was monitored via session logs, and exertion levels were controlled using the modified Borg scale after each session. Participants were encouraged to maintain a moderate perceived exertion level (3–5 on a 10-point scale) to ensure safe and effective training intensity appropriate for older adults.

The intervention consisted of a combined program incorporating traditional gerontogymnastics with a targeted protocol designed to improve muscular strength, balance, functional mobility and proprioception. Sessions followed a structured progression tailored to participant needs. Each session was delivered in a controlled environment, over 6 weeks [51], with two 45 min sessions per week [21,52]. Adherence was high across all groups, with participants completing all 12 scheduled sessions (100% adherence rate). This level of consistency strengthened the internal validity of the intervention effects.

**Figure 2:**
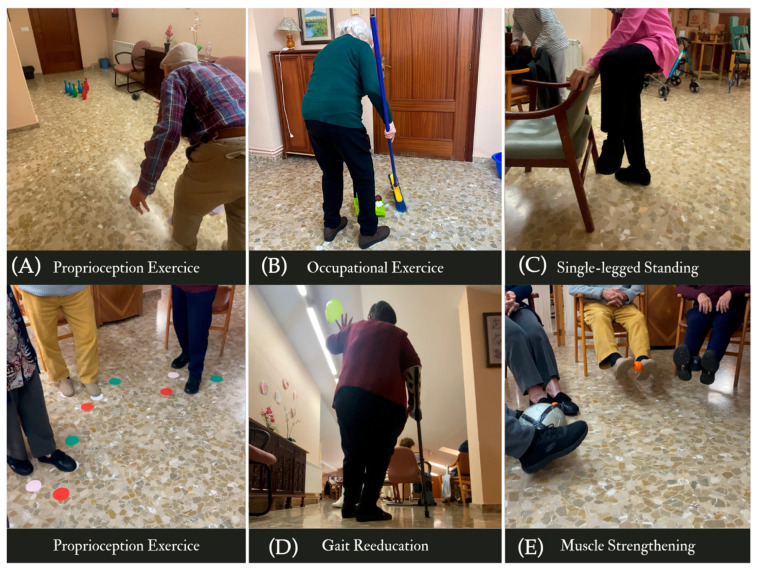
Multicomponent Occupational Therapy Intervention. This figure illustrates core activities used in the multicomponent program, categorized by their therapeutic goals: (**A**) Proprioception exercises: Balance tasks involving visual occlusion, unstable surfaces, or precise foot placement to improve spatial awareness and joint position sense (e.g., bowling while standing on foam, ‘Twister’-style stepping games). (**B**) Occupational exercise: Functional tasks simulating real-life activities, such as sweeping or object collection, to enhance occupational performance and lower-limb coordination. (**C**) Single-leg standing: Static balance exercise aimed at improving postural stability and ankle strategy. (**D**) Gait reeducation: Dynamic activities like balloon walking to promote rhythmic movement, weight shifting, and safe ambulation. (**E**) Muscle strengthening: Resistance tasks using balls or bands to target lower-limb strength (e.g., leg lifts with ball, knee squeezes) to support functional mobility.

- IG1: Received a conventional gerontogymnastics program. The conventional gerontogymnastics program followed general guidelines used in Spanish long-term care centers. It consisted of low-impact exercises aimed at maintaining joint mobility, muscular flexibility, aerobic capacity, and general motor coordination. Sessions included warm-up routines, rhythmic seated and standing movements, and relaxation techniques, with adaptations based on individual capacity. Warm-up routines consisted of 5–7 min of joint mobilization (e.g., neck rolls, shoulder circles), breathing control exercises, and light dynamic stretching to prepare participants physically and cognitively. This type of intervention is widely used in geriatric settings to promote physical maintenance and prevent sedentarism in older adults [22].
- IG2: Received a multicomponent program (Figure 2) that included gerontogymnastics and targeted components aimed at:
  ○ Muscle Strengthening: Dynamic exercises using weights or resistance bands targeting the gluteus maximus, hamstrings, and quadriceps (seated and standing squats, heel lifts with trunk control, standing leg lifts and foot circles).
  ○ Balance Training: Both static and dynamic tasks, including bowling—a meaningful occupational activity within the leisure domain—and walking exercises to improve functional mobility.
  ○ Coordination Exercises: Tasks such as throwing balls or manipulating objects while walking, designed to enhance functional relevance and motor planning. Marching in place and distractive gait (e.g., walking while tossing a balloon), sit-to-stand with a ball toss.
  ○ Proprioceptive Training: Exercises aimed at increasing spatial awareness and body positioning. Exercises included balance tasks on foam pads, tandem stance with head turns, and closed-eye postural tasks such as single-leg stance or seated perturbation recovery. Additionally, participants practiced joint angle reproduction with visual occlusion using forward lean replication tasks. Further proprioceptive stimulation included activities such as: (a) Ball transfers above and in front of the head (arms in cross position); (b) Lateral trunk inclinations while transferring a ball across the body; Ball passes with the feet and in a figure-eight pattern between legs; (c) Balance on one leg with ankle strategy reinforcement; (d) Heel and toe raises with eyes closed; (e) ‘Twister’-style foot placement games for direction and weight shifting.

These exercises were chosen for their ability to enhance sensorimotor integration, challenge dynamic equilibrium, and stimulate joint-position awareness.

The intervention followed a restorative approach, aiming to recover performance skills like postural stability and balance, and a preventive approach focused on reducing disability risk associated with aging. The sessions also incorporated occupation-centered activities (e.g., leisure-based bowling) and preparatory methods to develop specific occupational performance skills.

Each exercise was performed in two sets of 12 repetitions, following evidence-based guidelines for optimal repetition in older populations [32]. Additional tasks targeted lower limb strengthening, upper limb joint mobility, and both static and dynamic balance through proprioceptive stimulation and postural stability. Exercise intensity was monitored using the modified Borg Rating of Perceived Exertion scale, recorded immediately after each session to capture overall perceived effort. The target range was 3–5 (‘moderate intensity’), consistent with recommendations for frail older adults. In addition, exercise parameters such as number of repetitions, sets, and range of motion were adjusted individually to maintain participants within this perceived exertion window. Although no objective physiological markers (e.g., heart rate, load monitoring) were used for each exercise, the sessions were supervised by trained occupational therapists who adapted the difficulty according to tolerance and safety. This pragmatic approach reflects the clinical context of institutionalized older adults, where safety and feasibility often take precedence over maximal intensity control. We acknowledge this as a methodological limitation and recommend that future trials incorporate objective measures of exercise intensity to enhance prescription accuracy and reproducibility.

### 2.6. Statistical Analysis

Statistical analyses were conducted using Jamovi software Version 2.3.28.0. Analyses were performed on a complete-case basis due to the absence of dropouts. All participants completed the intervention and both pre- and post-intervention assessments, allowing a complete-case analysis without the need for imputation or dropout adjustments.

Normality of data distribution was tested using the Shapiro–Wilk test, and homogeneity of variances was assessed with Levene’s test. Descriptive statistics were expressed as mean ± standard deviation. To assess the impact of the intervention, a two-way mixed ANOVA was used for each primary and secondary outcome, with time (pre/post) as the within-subject factor and group (IG1, IG2, CG3) as the between-subject factor. Group × time interactions were analyzed using mixed two-way ANOVA. Where appropriate, effect sizes (partial η^2^) were calculated, and pairwise comparisons were adjusted using Tukey’s post hoc test.

Post hoc analysis (Tukey’s HSD) was conducted only in cases where significant interaction effects were observed. Effect sizes were interpreted using partial eta squared (η*p*^2^) with the following conventional thresholds: small (0.01 ≤ η*p*^2^ < 0.06), medium (0.06 ≤ η*p*^2^ < 0.14), and large (η*p*^2^ ≥ 0.14).

All tests were two-tailed, with significance set at *p* < 0.05. Due to the exploratory and pilot nature of the trial, results are interpreted as preliminary.

## 3. Results

### 3.1. Baseline Characteristics

The demographic characteristics were as follows: IG1: Age 83.3 ± 9.19 years, body mass 63.2 ± 12.5 kg, height 158 ± 9.24 cm; IG2: Age 87.0 ± 5.94 years, body mass 66.7 ± 12.2 kg, height 159 ± 7.63 cm; CG3: Age 88.1 ± 9.32 years, body mass 61.0 ± 10.8 kg, height 160 ± 10.4 cm.

No statistically significant differences were observed between groups in age (*p* = 0.418), height (*p* = 0.904), body mass (*p* = 0.522), baseline pain (VAS; *p* = 0.544), cognitive status (MMSE; *p* = 0.245), proprioceptive error (JPS; *p* = 0.266), or perceived exertion (Borg scale; *p* = 0.469). However, significant differences were found in baseline values for Timed Up and Go (TUG; *p* = 0.035), POMA (*p* = 0.049), and the Functional Independence Measure (FIM; *p* = 0.006), indicating that the groups were not entirely homogeneous in functional status at baseline (Table 1).

Specifically, the CG3 showed the poorest initial performance in the TUG (47.0 ± 23.7 s). IG2 presented intermediate results (32.2 ± 13.2 s), while IG1 was the fastest (25.1 ± 8.61 s). A similar pattern was observed in the POMA scores, with CG3 scoring the lowest (13.4 ± 6.21) compared to IG1 (19.4 ± 2.69) and IG2 (19.5 ± 4.61). In terms of FIM, CG3 also showed reduced functional independence (90.1 ± 12.3) relative to IG1 (107 ± 19.4) and IG2 (110 ± 13.0).

### 3.2. Primary Outcomes (Table 2)

- *Mobility (TUG)*

No significant group × time interaction was observed for TUG performance (F (2, 32) = 0.30, *p* = 0.744, η*p*^2^ = 0.018). Across all groups, descriptive results indicated a general decline over time, most marked in CG3 (+11.7 s).

- *Balance (POMA).*

The interaction effect did not reach statistical significance (F (2, 32) = 2.76, *p* = 0.082, η*p*^2^ = 0.147). IG2 maintained stable scores (19.5 ± 4.61 to 19.6 ± 3.80), whereas IG1 (19.4 ± 2.69 to 17.0 ± 3.79) and CG3 (13.4 ± 6.21 to 12.4 ± 5.50) showed a decline.

- *Functional Independence (FIM).*

No significant interaction was detected (F (2, 32) = 0.32, *p* = 0.728, η*p*^2^ = 0.020). IG2 preserved functional independence (110 ± 13.0 pre and post), while IG1 (107 ± 19.4 to 105 ± 18.8) and CG3 (90.1 ± 12.3 to 87.6 ± 13.0) showed a tendency toward deterioration.

- *Proprioception (JPS).*

No significant interaction was observed (F (2, 32) = 2.77, *p* = 0.082, η*p*^2^ = 0.145). Descriptively, IG2 (9.23 ± 8.13 to 5.69 ± 4.05) improved proprioceptive accuracy (−3.54°), while IG1 worsened (9.54 ± 8.09 to 11.70 ± 7.22). In this sense, IG2 improved proprioception with a mean reduction of 3.54° in JPS error, approaching the MCID threshold of 2°.

### 3.3. Secundary Outcomes

Significant group × time interactions were observed for muscle strength of the right quadriceps and hamstring (F (2, 32) = 5.94, *p* = 0.006, η*p*^2^ = 0.271) and for perceived exertion (Borg scale) (F (2, 32) = 4.79, *p* = 0.015, η*p*^2^ = 0.230). Post hoc comparisons revealed that both intervention groups (IG1 and IG2) preserved or improved muscle strength significantly more than the control group (CG3). Additionally, both intervention groups reported reduced perceived exertion levels post-intervention compared to CG3 (Table 2).

### 3.4. Clinical Relevance

Although most interaction effects for primary outcomes did not reach statistical significance, descriptive trends suggested potential clinical relevance. Based on established MCID thresholds (TUG ≥ 1.3 s, POMA ≥ 3 points, JPS ≥ 2° improvement, FIM ≥ 5 points), IG2 demonstrated favorable changes in proprioception and functional independence, while IG1 and CG3 showed declines. These results, although exploratory, align with the hypothesis that multicomponent interventions may help preserve neuromuscular function in institutionalized older adults.

**Figure 3:**
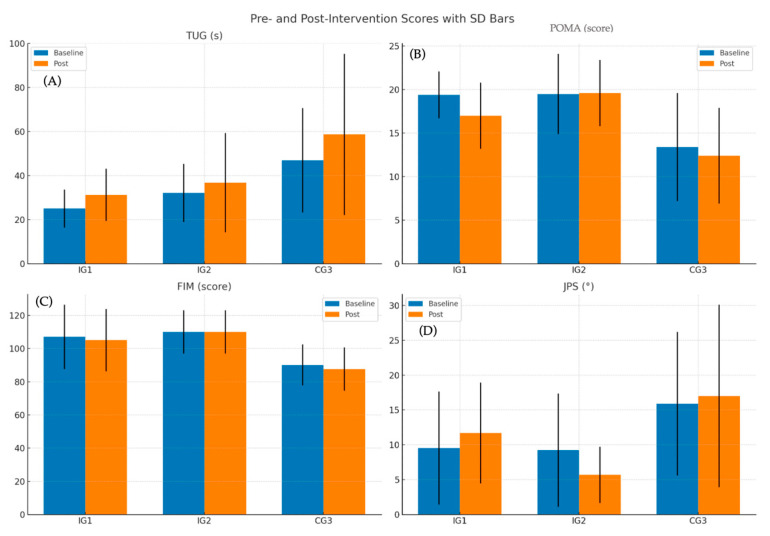
Pre–Post-Intervention Changes by Group. Line graphs illustrating changes from baseline to post-intervention across the three study groups: IG1 (gerontogymnastics program), IG2 (multicomponent intervention), and CG3 (control group). (**A**) Timed Up and Go (TUG): Higher values indicate slower mobility and greater fall risk. (**B**) Performance-Oriented Mobility Assessment (POMA): Lower scores reflect poorer balance and higher instability. (**C**) Functional Independence Measure (FIM): Higher scores indicate greater functional independence in daily activities. (**D**) Joint Position Sense (JPS): Higher values denote increased proprioceptive error. Each line represents group mean values at each time point; vertical error bars denote standard deviation (SD).

Graphical representations (Figure 3) illustrate these patterns. IG2 consistently showed maintenance or improvement across most outcomes, including proprioception, balance, and functional mobility. In contrast, IG1 and CG3 demonstrated greater variability and functional decline.

Although the differences in FIM scores were not statistically significant, IG2 preserved functional independence post-intervention, while IG1 and CG3 showed a decline. This trend aligns with clinical observations and highlights the potential utility of multicomponent interventions in maintaining autonomy in institutionalized older adults.

## 4. Discussion

The primary aim of this randomized controlled trial was to evaluate changes in balance, functional mobility, functional independence, and proprioceptive acuity among institutionalized older adults following a structured intervention that combined gerontogymnastics wfith proprioceptive and strength-based exercises. This approach sought to provide evidence on the effectiveness of targeted therapeutic programs in improving balance and functional capacity in aging populations. The integration of multicomponent strategies responds to the growing body of literature suggesting that unimodal interventions may be insufficient for addressing the multifactorial nature of functional decline in older adults [18].

Given the exploratory pilot nature and the small sample size, the statistical power was insufficient to detect modest effects. Therefore, all findings should be considered preliminary indicators of feasibility and clinical trends rather than definitive evidence of efficacy. Importantly, no post hoc or simple effect analyses were conducted in the absence of significant interaction or main effects, in order to avoid overinterpretation. To provide a comprehensive view, it is essential to interpret the results across all measured outcomes—balance, proprioception, functional mobility, muscle strength, and perceived exertion—as interconnected indicators of neuromuscular health and fall risk in older adults.

The results suggest a trend toward improved proprioception in the multicomponent group (IG2), approaching the MCID threshold (≥2° error reduction), although this did not reach statistical significance. This trend may suggest a potential protective effect against age-related functional decline, particularly in terms of balance and gait, which are critical predictors of fall risk and independence. However, further research is needed to confirm this effect. Moreover, pain levels remained stable in the intervention groups (IG1 and IG2), whereas the control group (CG3) reported increased pain perception post-intervention. Although the difference was not statistically significant, the clinical relevance lies in the observed trend of deterioration in CG3, which supports the pain-adaptation model. This model posits that persistent pain may lead to reduced physical activity, resulting in muscle disuse, weakness, and progressive discomfort [53]. In this context, physical inactivity exacerbates functional decline and contributes to a negative cycle of decreased mobility and increased pain. This pattern has also been described in studies of musculoskeletal aging where movement limitation, progressive sarcopenia and negative perception of effort coexist, favoring isolation and loss of functional autonomy [51].

Regarding the TUG test, all groups exhibited performance decline, with the most marked deterioration in IG1 and CG3. These findings may reflect the natural progression of aging-related functional decline, particularly in the absence of specific neuromuscular stimuli [54,55]. IG2, however, showed the smallest reduction in performance, indicating that the multicomponent intervention may buffer the expected decline, possibly due to its targeted approach combining strength, proprioception, and balance elements [18]. It should be noted, however, that the TUG test has limited predictive validity for falls when used in isolation, as highlighted in Barry et al. [34]. Therefore, our interpretation emphasizes mobility performance rather than direct fall prediction, reinforcing the need to consider TUG findings alongside balance, proprioception, and functional independence outcomes. Multicomponent training involving proprioception, dynamic balance, and resistance exercises can stimulate sensory feedback loops and motor planning, enhancing sensorimotor integration and neuromuscular control—key elements in maintaining postural stability in older adults. From a physiological standpoint, the observed trends can be interpreted through the lens of sensorimotor plasticity and neuromuscular adaptation. Multicomponent interventions that include proprioceptive stimulation, balance challenges, and strength training promote reweighting of sensory inputs (visual, vestibular, and somatosensory), which is critical in aging populations due to declining sensory acuity. Enhanced proprioceptive feedback contributes to better joint position awareness, while resistance training activates type II muscle fibers, which are preferentially lost with age. These adaptations support improved motor planning and stability during dynamic tasks, ultimately aiding in fall prevention. Furthermore, repetitive functional movement can enhance cortical representation of motor patterns, contributing to neuroplastic changes that support balance and coordination [30,31,56,57]. These findings, although preliminary, reinforce the importance of targeting multiple physiological systems simultaneously, as deterioration in one domain (e.g., proprioception) may compromise overall mobility and increase fall risk even when muscle strength is preserved. These mechanisms are supported by evidence showing that combining balance, strength, and proprioceptive training enhances corticospinal excitability and sensory reweighting in older adults, leading to improved gait stability and reaction to perturbations [29,58].

Fall risk analysis via the POMA revealed a significant decline in IG1 (*p* < 0.05), whereas IG2 maintained stable scores. CG3 also declined, though without statistical significance. Notably, IG2 achieved a mean POMA score of 19.6 ± 3.80 post-intervention, compared to 17 ± 3.79 in IG1 and 12.4 ± 5.50 in CG3. These findings suggest that gerontogymnastics alone may be less effective than multicomponent interventions in addressing key aspects of balance, though this requires confirmation in adequately powered studies. The superior outcomes in IG2 align with previous studies supporting the use of multicomponent programs for enhancing postural stability and reducing fall risk in older populations [31,59]. The observed improvements in TUG and POMA in IG2 approached or exceeded MCID thresholds (≥1.3 s and ≥3 points, respectively), which may indicate potential clinical relevance, although statistical significance was not reached. Comparative evidence from Li et al. [23], who implemented a Tai Chi-based intervention, found significant improvements in balance and reduced fall incidence over six months. While our study used different methods and a shorter duration, the positive trends observed in IG2 suggest that diverse multimodal interventions can yield comparable benefits when appropriately tailored.

The maintenance or improvement of functional independence across intervention groups also appear consistent with changes in pain, proprioception, and exertion tolerance, supporting the hypothesis that neuromuscular activation and sensorimotor feedback are key mediators of autonomy in daily tasks. Functional independence, measured through the FIM scale, declined in CG3, remained stable in IG2, and slightly deteriorated in IG1. Maintaining FIM scores aligns with findings by Minobes-Molina et al. (2023), who reported that combining physical and coordination tasks prevents decline in ADL performance compared to exercise alone [20]. These variations suggest that physical activity plays a vital role in preserving ADL performance among institutionalized seniors. The decline in IG1 may be explained by the lack of targeted exercises for coordination and balance, which are essential for executing complex tasks. In contrast, IG2′s inclusion of proprioceptive and coordination tasks likely enhanced neuromuscular integration, supporting functional autonomy [31,59]. These variations suggest that physical activity plays a vital role in preserving ADL performance among institutionalized seniors. Notably, motor learning principles such as task specificity and repetition are essential for maintaining independence in this population. By engaging in occupation-based activities that simulate real-life tasks (e.g., bowling, ball-passing), IG2 participants likely benefited from improved transfer of training, which reinforces functional autonomy [60,61].

Proprioceptive accuracy, as assessed by JPS, worsened significantly in IG1 (*p* < 0.05) but improved clinically in IG2, although without statistical significance, exceeding the MCID threshold of ≥2° error reduction. This trend may still be considered clinically meaningful as it aligns with previously reported thresholds for relevant change in proprioception among older adults, particularly when coupled with improved balance performance [62].

The divergent trends observed across outcome measures may be attributed to multiple factors. First, the duration and intensity of the intervention may have been sufficient to elicit proprioceptive and static balance improvements, but not strong enough to induce significant neuromuscular adaptations required for dynamic gait tasks such as those evaluated in the TUG. Moreover, tasks requiring cognitive-motor integration or rapid force production may benefit from longer or more intense training. Second, the specificity of training likely played a role—IG2 proprioceptive and balance challenges more closely align with postural control than with complex gait reorganization. Lastly, ceiling effects in certain scales and baseline functional heterogeneity may have influenced the magnitude of observed changes.

The Borg scale revealed that perceived exertion increased in CG3, while it remained stable in the intervention groups. This suggests that regular physical engagement helps maintain exertion tolerance and mitigates fatigue perception, which is closely linked to cardiovascular and muscular conditioning [13,48,63,64]. These findings may reflect improved sensorimotor efficiency and cardiovascular conditioning in the intervention groups, highlighting the integrative effect of multicomponent training on both perceived and objective physical function [13,65]. This aligns with studies demonstrating that moderate multicomponent exercises maintain aerobic fitness and perceived exertion levels by improving metabolic efficiency and central fatigue thresholds [17].

Overall, the trends observed across multiple domains suggest that multicomponent, occupation-based interventions may generate beneficial effects that address not only physical but also perceptual and motivational aspects of aging. These interconnected gains—though not always statistically significant—offer clinically meaningful insights into how integrative therapy can preserve mobility, reduce perceived fatigue, and support independence in institutionalized older adults. The discussion and interpretation of results must be framed within the constraints of a small sample size and pilot design. Although positive trends were observed, they should serve to inform the design of future, larger-scale studies rather than establish clinical recommendations.

### 4.1. Practical Implications

The findings of this study have direct applications in geriatric care and occupational therapy. Incorporating multicomponent programs that integrate strength, balance, and proprioception training into routine care can enhance physical autonomy and reduce fall risk among institutionalized older adults. These interventions are feasible to implement within long-term care settings using minimal resources and can be customized based on residents’ functional levels. Furthermore, occupational therapists may use these results to design more holistic intervention strategies that align with clients’ meaningful occupations and real-life activities.

### 4.2. Future Research Directions

Future studies should aim to replicate these findings using larger, more heterogeneous samples across different care contexts. Longitudinal studies with extended intervention durations and follow-up periods are needed to assess the sustainability and long-term impact of these programs. In addition, future research may explore the dose–response relationship between training intensity and outcomes, the role of cognitive-motor tasks in enhancing neuromuscular performance, and the integration of digital or wearable technology for proprioceptive feedback. From an occupational therapy perspective, the results obtained reinforce the need to apply occupation-focused interventions that integrate functional training, neuromuscular stimulation and meaningful activities for users. The incorporation of activities with occupational value such as bowling or functionally loaded walking exercises demonstrates that it is possible to intervene in key areas of occupational performance through a structured, restorative, and preventive approach, aligned with models such as the Model of Human Occupation (MOHO) and User-Centered Practice. It is also recommended to explore the integration of dual cognitive-motor demand tasks, as well as the use of accessible technology (such as motion sensors or mobile apps) to improve proprioceptive feedback and long-term adherence. Finally, one limitation of this study is that randomization was not stratified by sex, which may influence neuromuscular and balance-related outcomes. Although the gender distribution across groups was relatively balanced (with slightly more females in each group), future studies should consider sex as a stratification variable or covariate in the analysis, as gender differences in muscle strength, gait dynamics, and postural control have been reported in geriatric populations [57].

### 4.3. Limitations

The small sample size is a major limitation, and the study was underpowered to detect modest between-group effects. No formal power analysis was conducted prior to recruitment, as this was a pilot feasibility trial intended to assess preliminary effects and implementation feasibility. Therefore, the findings should be considered exploratory. Future studies should include larger samples based on power calculations derived from the effect sizes observed here.

Analyses were limited to main and interaction effects from the mixed ANOVA. Post hoc tests were only applied where interaction effects were statistically significant, consistent with best statistical practice. The lack of significant pairwise differences reflects both the exploratory nature of this pilot and the limited sample size. Another key limitation is the relatively short duration of the intervention (6 weeks). While similar timeframes have demonstrated feasibility and initial benefits in comparable populations [21], it is plausible that a longer intervention could have led to more robust and sustained improvements, particularly in balance, proprioception, and muscle strength. Additionally, the lack of long-term follow-up prevents assessment of the durability of the observed gains. Although short-term improvements were detected in several outcomes, it remains unclear whether these changes persist in the absence of continued therapeutic activity. Future studies should replicate this design with larger and more heterogeneous samples, longer intervention periods (e.g., 8–12 weeks), and follow-up assessments to determine the long-term efficacy and functional impact of multicomponent occupational interventions in institutionalized older adults.

Another methodological limitation is the lack of direct neurophysiological measures of postural control, such as posturography or center-of-pressure analysis. While our study evaluates functional balance and proprioceptive accuracy, these assessments reflect performance-level outcomes rather than underlying neural control processes. Finally, this study evaluated fall risk indirectly through functional and balance measures. Actual fall incidents were not recorded during or after the intervention period, which limits conclusions on real-world fall prevention.

## 5. Conclusions

Statistically significant improvements were observed in lower-limb muscle strength—specifically in the right quadriceps and hamstrings—in the multicomponent group (IG2), indicating short-term neuromuscular benefits. In terms of proprioception and balance, IG2 showed improvements that approached established MCID thresholds, although these did not reach statistical significance. No significant changes were observed in pain perception or perceived exertion across groups.

Regarding mobility and fall risk, IG2 was the only group to maintain functional performance in the TUG and POMA scores, while both IG1 and CG3 showed declines. Although these changes were not statistically significant, their alignment with clinical relevance thresholds suggests the intervention may hold promise.

These preliminary findings suggest that multicomponent occupational therapy interventions combining proprioception, balance, and strength training may offer functional protection against age-related decline. However, due to the limited sample size and short intervention period, the results should be interpreted cautiously. Future studies should confirm these effects in larger, stratified samples with extended follow-up periods. Importantly, conclusions are based strictly on interaction and main effects, and exploratory clinical trends are reported without further subgroup testing to avoid misinterpretation. Thus, these findings should be viewed as preliminary signals that warrant confirmation in larger trials.

## Figures and Tables

**Table 1:** Baseline sociodemographic and clinical characteristics by group. Values are presented as mean ± standard deviation (SD). *p*-values were derived from one-way ANOVA.

Parameters	IG1 (Mean ± SD)	IG2 (Mean ± SD)	CG3 (Mean ± SD)	Total Sample (Mean ± SD)	*p* Value (ANOVA) *
Age (years)	83.3 ± 9.19	87.0 ± 5.94	88.1 ± 9.32	85.9 ± 8.19	0.418
Height (cm)	158 ± 9.24	159 ± 7.63	160 ± 10.4	159 ± 8.76	0.904
Body mass (kg)	63.2 ± 12.5	66.7 ± 12.2	61.0 ± 10.8	63.9 ± 11.9	0.522
VAS (score)	1.38 ± 1.26	1.00 ± 1.35	1.67 ± 1.41	1.31 ± 1.32	0.544
MMSE (score)	20.8 ± 4.74	20.5 ± 6.04	17.4 ± 4.61	19.8 ± 5.28	0.245
TUG (seconds)	25.1 ± 8.61	32.2 ± 13.2	47.0 ± 23.7	33.4 ± 17.2	0.035 *
POMA (score)	19.4 ± 2.69	19.5 ± 4.61	13.4 ± 6.21	17.9 ± 5.11	0.049 *
FIM (score)	107 ± 19.4	110 ± 13.0	90.1 ± 12.3	104 ± 17.2	0.006 *
JPS (°)	9.54 ± 8.09	9.23 ± 8.13	15.9 ± 10.3	11.1 ± 8.94	0.266
Borg scale (score)	2.10 ± 2.73	1.57 ± 1.56	2.97 ± 3.16	2.12 ± 2.48	0.469

MMSE: Mini-Mental State Examination; TUG: Timed Up and Go; FIM: Functional Independence Measure; JPS: Joint Position Sense; POMA: Performance-Oriented Mobility Assessment; cm: centimeters; kg: kilograms; SD: standard deviation; IG1: intervention conventional gerontogymnastics program; IG2: intervention multicomponent program; CG3: control group; * *p* < 0.05: statistically significant.

**Table 2:** Pre- and post-intervention values for primary and secondary outcomes across groups. Values are presented as mean ± standard deviation (SD). Two-way mixed ANOVA results are shown for main effects of interaction effect (time × group), with F-statistics, *p*-values, and partial eta squared (η*p*^2^). Post hoc (Tukey HSD) was performed only where interaction effects were significant. *p* < 0.05 indicates statistical significance.

	Variable	Group	Pre Intervention Mean ± SD	Post Intervention Mean ± SD	Interaction Effect (Time * Group Interaction)	
					F(*p*)	η*p*^2^
*Primary outcomes*						
Mobility	TUG (seconds)	IG1	25.10 ± 8.61	31.30 ± 11.80		0.018
		IG2	32.20 ± 13.20	36.80 ± 22.50	0.30 (0.744)	
		CG3	47.00 ± 23.70	58.70 ± 36.60		
Balance	POMA (score)	IG1	19.40 ± 2.69	17.00 ± 3.79		0.147
		IG2	19.50 ± 4.61	19.60 ± 3.80	2.76 (0.082)	
		CG3	13.40 ± 6.21	12.40 ± 5.50		
Functionality	FIM (score)	IG1	107.00 ± 19.40	105 ± 18.80		0.020
		IG2	110.00 ± 13.00	110 ± 13.00	0.32 (0.728)	
		CG3	90.10 ± 12.30	87.6 ± 13.00		
Proprioception	JPS (°)	IG1	9.54 ± 8.09	11.70 ± 7.22		0.145
		IG2	9.23 ± 8.13	5.69 ± 4.05	2.77 (0.082)	
		CG3	15.90 ± 10.30	17.00 ± 13.10		
Secondary outcomes						
Muscle strength outcomes	Right Quadriceps Strength	IG1	4.62 ± 0.506	4.62 ± 0.506	5.94 (0.006)	0.271
		IG2	4.62 ± 0.506	4.62 ± 0.506		
		CG3	4.11 ± 0.601	3.78 ± 0.667		
	Right Hamstring Strength	IG1	4.69 ± 0.480	4.69 ± 0.480	5.94 (0.006)	0.271
		IG2	4.62 ± 0.506	4.62 ± 0.506		
		CG3	4.00 ± 0.707	3.67 ± 0.500		
Perceived exertion	Borg Scale	IG1	2.10 ± 2.73 2	2.10 ± 2.73	4.79 (0.015)	0.230
		IG2	1.57 ± 1.56	1.57 ± 1.56		
		CG3	2.97 ± 3.16	5.04 ± 3.58		

SD: standard deviation; IG1: intervention conventional gerontogymnastics program; IG2: intervention multicomponent program; CG3: control group; F: Fisher’s *p*: *p*-value; η*p*^2^ = partial eta squared; TUG: Timed Up and Go; FIM: Functional Independence Measure; JPS: Joint Position Sense; POMA: Performance-Oriented Mobility Assessment.

## Data Availability

The data presented in this study are available on request from the corresponding author. The data are not publicly available due to privacy and ethical restrictions involving residents of a long-term care institution.
